# Systematic Review of Artificial Intelligence Applications in Clinical Trials for Central Nervous System Injuries

**DOI:** 10.2174/011570159X443258251114091930

**Published:** 2025-11-17

**Authors:** Xiangyu Lu, Xiao Xiao, Yilei Wu, Yonggang Wei, Bo Li

**Affiliations:** 1 Department of General Surgery, Division of Liver Surgery, West China Hospital, Sichuan University, Chengdu, Sichuan, 610041, China;; 2 Department of General Surgery, Division of Hepatobiliary-Pancreatic Surgery, Sichuan Provincial People's Hospital, University of Electronic Science and Technology of China, Chengdu, Sichuan, 610072, China;; 3 Department of Medical Genetics, West China Second University Hospital, Sichuan University, Chengdu, Sichuan, 610041, China;; 4 Key Laboratory of Birth Defects and Related Diseases of Women and Children (Sichuan University), Ministry of Education, Chengdu, Sichuan, 610041, China;; 5 Department of Medical Records Statistics, Sichuan Provincial People's Hospital, University of Electronic Science and Technology of China, Chengdu, Sichuan, 610072, China

**Keywords:** Artificial intelligence, central nervous system injuries, AI-driven approaches, convolutional neural networks (CNN), patient-reported outcome measures (PROMs), AI interventions

## Abstract

**Introduction:**

Artificial intelligence (AI) has emerged as a promising tool for diagnosing, managing, and treating the injuries of the central nervous system (CNS). The purpose of this study was to evaluate the AI-driven approaches in clinical trials for CNS diseases.

**Methods:**

A systematic search of the ClinicalTrials.gov, PubMed, Ovid, and Web of Science databases was conducted to identify interventional trials focusing on CNS injuries. Only interventional studies investigating AI applications in CNS injuries were included, while those targeting neurodegenerative diseases were excluded. Data extraction was performed using a self-designed form.

**Results:**

A total of 51 AI-driven clinical trials for CNS injuries were identified. Most trials focused on screening, diagnosis, monitoring, supportive care, prevention, and treatment, and were primarily conducted in China, the USA, and Europe. The targeted conditions included stroke and its sequelae, traumatic brain injury, and spinal cord injury. All trials employed AI-based tools supported by diverse algorithms, such as convolutional neural networks (CNN), extreme gradient boosting (XGBoost), and K-nearest neighbors (KNN). However, only 21.6% (11/51) of the trials reported outcome data, with 10 demonstrating functional improvements, mainly in motor, swallowing, and neurological performance. Notably, 25.5% of the trials incorporated patient-reported outcome measures (PROMs).

**Discussion:**

This study demonstrates the growing application of AI in CNS injury management, particularly in diagnosis and treatment. However, the limited reporting of outcomes and underuse of PROMs suggest that most interventions remain in early stages of clinical translation. Standardized trial designs, patient-centered measures, and rigorous performance validation will be essential to ensure the meaningful integration of AI into clinical practice.

**Conclusion:**

AI-based clinical trials for CNS injuries are on the rise, with a focus on diagnosis and treatment. Future trials should prioritize standardized designs, integration of PROMs, and comprehensive performance metrics to ensure clinically meaningful evaluation of AI interventions.

## 1 INTRODUCTION

Central nervous system (CNS) injuries, including traumatic brain injury (TBI), spinal cord injury (SCI), and stroke, frequently result in irreversible neurological deficits and impose a significant economic burden due to the high cost of medical care [1-18]. In 2019, an estimated 27.16 million new cases of TBI, 0.9 million new cases of SCI, and 12.2 million new cases of stroke occurred worldwide, resulting in approximately 7.08 million, 6.2 million, and 143 million disability-adjusted life-years, respectively [19-21]. Given the devastating impact and rising incidence of CNS injuries, accurate measurement and comprehensive monitoring of neurological function and treatment efficacy are essential for optimizing patient care and guiding clinical decision-making.

Artificial intelligence (AI) technologies have garnered increasing global attention, particularly in health care, where they are driving significant advancements [22-41]. In the field of CNS diseases, AI has emerged as a promising tool for diagnosis, management, and treatment [42-44]. By using machine learning algorithms, deep learning models, and natural language processing, AI can efficiently analyze large-scale clinical data, neuroimaging, and biomarkers to enhance diagnostic accuracy and predict patient outcomes [45-50]. In TBI, SCI, and stroke, AI-driven systems are used for early detection, injury severity classification, and prognostic assessment [51-54]. Additionally, AI-powered clinical decision support systems facilitate personalized treatment planning by integrating patient-specific data, while AI-assisted rehabilitation platforms optimize recovery monitoring and therapy adjustment [55, 56]. Therefore, the integration of AI into CNS disease management holds potential to improve clinical efficiency, enhance patient care, and accelerate the development of novel therapeutic strategies [57, 58].

Several reviews in recent years have examined the applications of AI in CNS diseases, highlighting its potential in CNS injury diagnosis, prognostic modeling, neurorehabilitation, and remote care [59-63]. Clinical trial evidence, however, remains the “gold standard” for rigorously evaluating the safety and efficacy of AI interventions under controlled conditions and for guiding regulatory approval [64]. To date, no comprehensive evaluation of AI-driven approaches in clinical trials for CNS diseases has been reported. A systematic review is therefore warranted to assess the effectiveness, challenges, and future directions of AI in this field. This review systematically analyzes the current landscape of AI-based clinical trials and assesses their impact on treatment outcomes in CNS injuries.

## 2 METHODS

### 2.1 Search Strategy

We selected records from the ClinicalTrials.gov registry, Pubmed, Ovid, and Web of Science using the following algorithm: (“Artificial Intelligence” OR “AI” OR “Machine Learning” OR “ML” OR “Deep Learning” OR “DL” OR “Natural Language Processing ” OR “NLP” OR “Neural Network*” OR “Artificial Neural Network*” OR “Computer Vision” OR “Large Language Model” OR “LLM”) and “Central Nervous System Injuries” OR “CNS Injuries” OR “Traumatic Brain Injury” OR “TBI” OR “Concussion” OR “Diffuse Axonal Injury” OR “Intracranial Hemorrhage” OR “Brain Contusion” OR “Blast Injury” OR “Spinal Cord Injury” OR “SCI” OR “Stroke” OR “Ischemic Stroke” OR “Hemorrhagic Stroke” OR “Cerebral Hypoxia” OR “Subarachnoid Hemorrhage” OR “Hypoxic-Ischemic Encephalopathy” OR “HIE”. The trial search was conducted up to August 11, 2025, without any language limitations.

### 2.2 Eligibility Criteria

The inclusion criteria required interventional studies regardless of the trial status (*i.e*., not yet recruiting, recruiting, completed, terminated, enrolling by invitation, suspended, withdrawn, or unknown), the trial phase (*i.e*., early phase 1, phase 1, phase 2, phase 3, phase 4, or not applicable), and the trial results (*i.e*., with or without). We excluded observational studies as they provide lower-level evidence compared to interventional trials, and trials not assessing AI-enabled CNS injuries, as they were not relevant to the aim of this study.

### 2.3 Data Extraction

The retrieved data was saved in comma-separated values (CSV) format and converted into an Excel file. The following information was extracted: national clinical trial (NCT) number, study title, study status, study type, study design, study results, study phases, conditions, enrollment number, interventions, primary and secondary outcome measures, study record dates, and sponsor (Table **S1**).

### 2.4 Risk of Bias Assessment for the Included Trials

A self-designed scoring system was developed to assess the reporting quality and potential risk of bias for each included trial (Table **S2**). This system was informed by the framework proposed by Zarin *et al.* [65], which outlines key elements of registration quality and reporting transparency in clinical trials. Specifically, this review evaluated four items: recruitment status, availability of posted results, reporting of primary outcomes, and enrolled sample size. Each item was scored from 0 to 2, yielding a total score ranging from 0 to 8. Trials with a total score of 5 or below were classified as low quality, while those scoring above 5 were considered high quality.

### 2.5 Statistical Analysis

All data were imported into Microsoft Excel and analyzed using SPSS version 19.0 (SPSS Inc., Chicago, IL, USA). Descriptive statistics were used to summarize the characteristics of the included trials. The chi-square test was conducted to examine the association between the use of patient-reported outcome measures (PROMs) and the AI intervention objectives (*e.g*., diagnosis, treatment, and monitoring). Statistical significance was set at *P* < 0.05. Figures were created using GraphPad Prism version 8.0 (GraphPad Software, San Diego, CA, USA).

## 3 RESULTS

### 3.1 Eligible Clinical Trials

**Fig. (1):**
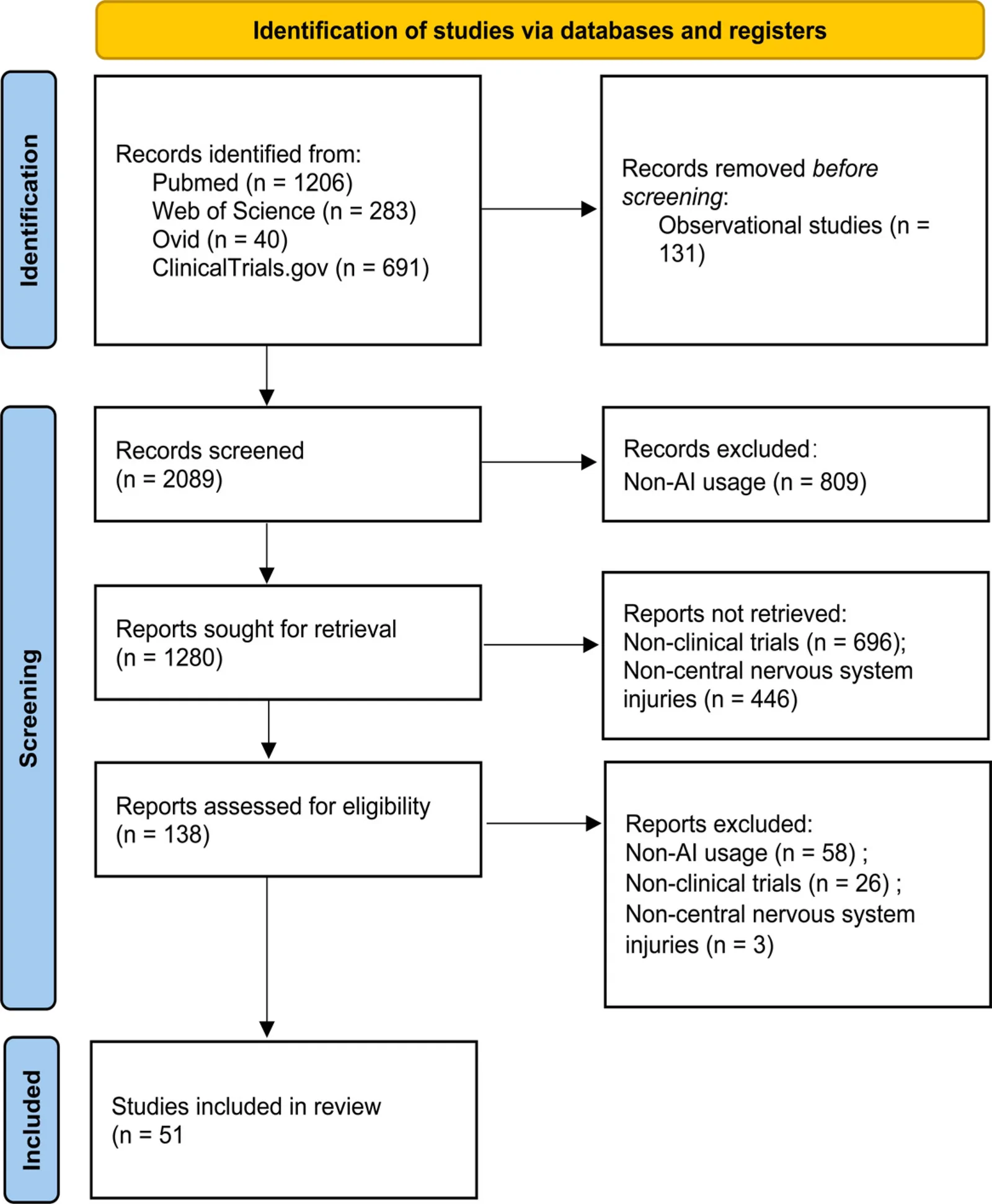
Flowchart illustrating the selection of Artificial intelligence clinical trials.

This study was conducted in accordance with the Preferred Reporting Items for Systematic Reviews and Meta-Analyses (PRISMA) 2022 guidelines. The search strategy yielded a total of 2,220 records, including 691 from ClinicalTrials.gov, 1,206 from PubMed, 40 from Ovid, and 283 from Web of Science. After excluding 131 observational studies, 2089 records were retrieved for title screening. Of these, 1951 were excluded for non-AI usage (n = 809), non-clinical trials (n = 696), and non-CNS injuries (n = 446). The remaining 138 records underwent a full-text assessment, among which 87 were excluded for reasons such as non-AI usage (n = 58), non-clinical trials (n = 26), and non-CNS injuries (n = 3). This resulted in the inclusion of 51 trials for final analysis (Fig. **1**).

### 3.2 Characteristics of Included Studies

**Fig. (2):**
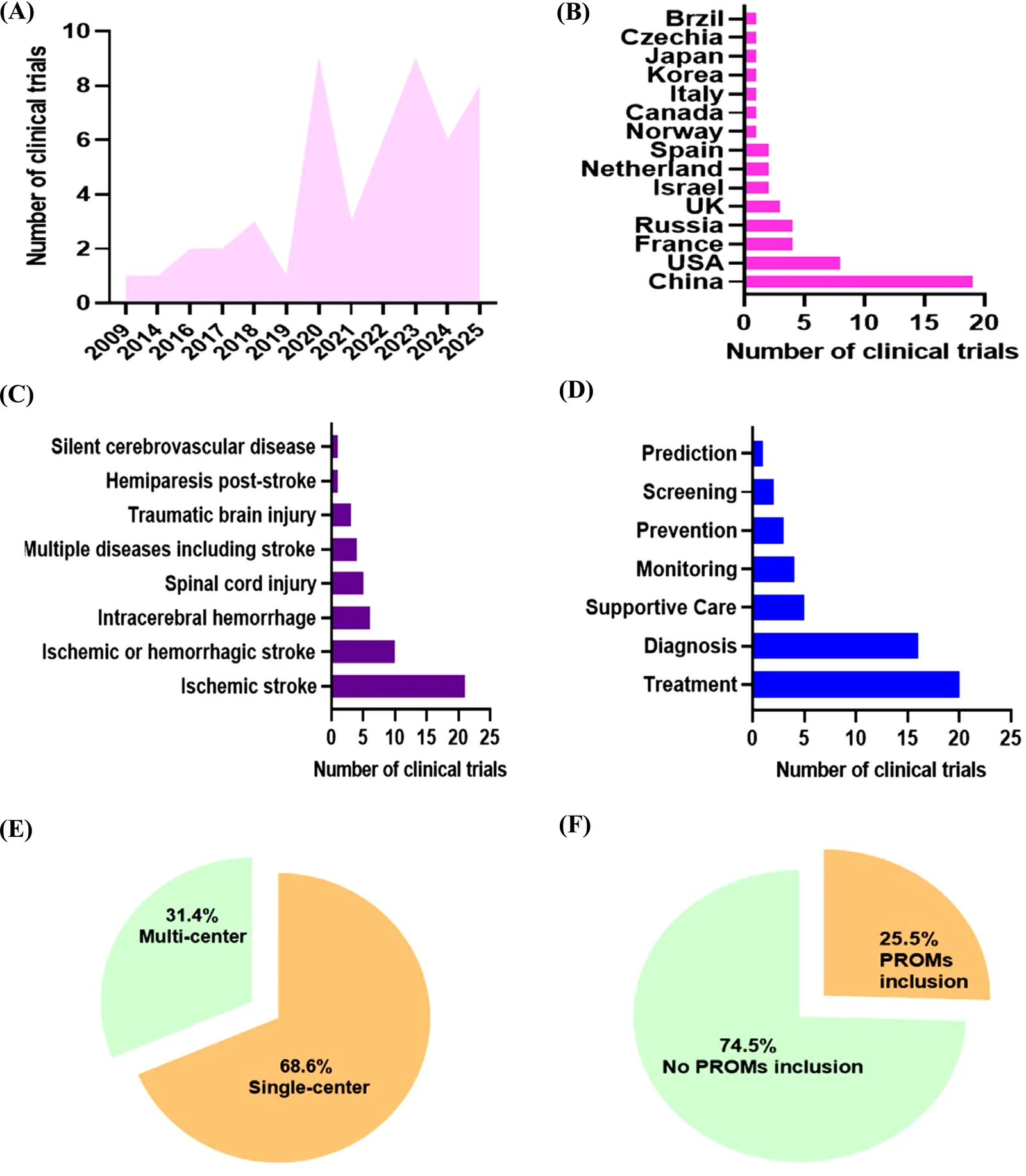
General characteristics of AI clinical trials included in this analysis. (**A**) Start dates of the clinical trials; (**B**) Countries conducting AI clinical trials; (**C**) Neurological disorders investigated in AI clinical trials; (**D**) Role of AI in the clinical trials; (**E**) Proportion of single-center and multi-center clinical Trials; (**F**) Proportion of AI clinical trials with and without PROMs.

Detailed information on the included clinical trials is summarized in Table **1**. The first AI-based clinical trial for CNS diseases emerged in 2009 [66]. By 2023, the number of trials had increased to nine per year. Considering that data collection extended only to August 2025, with eight trials already identified (Fig. **2A**), it is reasonable to note that the number of trials in 2025 is close to that in 2023, suggesting a continued upward trend in AI applications for CNS diseases.

Most AI clinical trials (37.3%) were conducted in China, followed by 15.7% in the USA, 7.8% in France and Russia, 5.9% in the UK, 3.9% in Netherland, Spain, and Israel, and 2.0% in Canada, Italy, Norway, Korea, Japan, Czechia, and Brazil (Fig. **2B**), underscoring the geographic concentration of AI clinical trial activities.

Based on disease type, the most common condition in these AI-related clinical trials was stroke and its sequelae, accounting for approximately 84.3% of the studies, followed by spinal cord injury (9.8%) and traumatic brain injury (5.9%), suggesting the potential of AI applications in managing severe neurological conditions (Fig. **2C**).

According to the functional role of AI in clinical trials, the studies were categorized into seven groups: 20 trials (39.2%) focused on treatment, 16 (31.4%) on diagnosis, 5 (9.8%) on supportive care, 4 (7.8%) on monitoring, 3 (5.9%) on prevention, and 2 (3.9%) on screening, and 1 (2.0%) on prediction (Fig. **2D**). Among the 51 clinical trials, 31.4% were multi-center studies, while 68.6% were conducted at a single center (Fig. **2E**). According to the risk of bias assessment, only 13 trials (25.5%) were rated as high quality. Notably, phase information was unavailable for most of the trials (49/51), reflecting limited methodological transparency in the clinical trials.

Almost all of the included clinical trials employed AI-based tools, such as AI-driven video-game systems, AI-powered virtual assistants, and AI-controlled robots. The algorithms primarily used in these trials included convolutional neural networks (CNN), random forests, logistic regression, extreme gradient boosting (XGBoost), K-nearest neighbors (KNN), AdaBoost, and gradient boosting machines (GBM). Outcome data were not reported in trials retrieved from ClinicalTrials.gov, whereas 11 of 15 trials identified from PubMed reported performance metrics, including accuracy, sensitivity, specificity, and area under the curve (AUC). Notably, 10 of these 11 trials demonstrated that AI interventions facilitated recovery from CNS disorders, underscoring the promising clinical applicability and translational potential of AI-based approaches in this field.

### 3.3 PROMs in AI Clinical Trials

PROMs are standardized tools used to capture patients’ perspectives on their health status, symptoms, and overall quality of life. They play a crucial role in clinical trials by providing valuable insights into the effectiveness and impact of interventions from the patient’s viewpoint [67-69]. Therefore, this study extracted PROMs data from AI clinical trials, as shown in Fig. (**2F**). The results revealed that only 13 trials (25.5%) incorporated PROMs, whereas 38 trials (74.5%) did not, indicating a notable underrepresentation of patient-centered outcomes in AI-based clinical research. Among the 13 clinical trials that incorporated PROMs, 10 (76.9%) were designed to evaluate therapeutic interventions, in contrast to 13 (34.2%) of the 38 trials without PROMs (*P* = 0.008). This significant association suggests a potentially stronger alignment between AI-based clinical research and therapeutic objectives through the integration of PROMs.

## 4 DISCUSSION

The analysis of AI-based clinical trials for CNS diseases shows a clear upward trend in recent years, reflecting the growing interest in AI applications in this field [70-75]. A key finding from this study was the geographic concentration of AI clinical trials, with the majority conducted in China. The USA, which followed China in contributing to AI clinical research, reflects its leadership in medical technology development [76]. The presence of trials in Europe, including those in France, the UK, Russia, Italy, Spain, Norway, and the Netherlands, demonstrates the broad international interest in AI applications for healthcare. However, no trials have been conducted in Africa, suggesting disparities in access to advanced medical interventions.

The predominance of Chinese trials indicates China’s robust investment in AI technologies, particularly within the healthcare sector. This surge is largely driven by national strategies, such as the “New Generation Artificial Intelligence Development Plan” [77], which promotes AI innovation, infrastructure development, and integration into clinical practice. China’s large population base, centralized medical data systems, and government support have created favorable conditions for the rapid deployment and testing of AI-based tools in clinical settings. However, this geographic concentration also raises concerns regarding the generalizability of the findings. AI models trained predominantly on Chinese patient data and healthcare systems may face limitations when applied to other populations due to variations in clinical workflows, disease patterns, genetic backgrounds, and sociocultural factors. To facilitate broader application, it is critical to conduct external validation studies in diverse geographic and healthcare contexts, including adapting and testing algorithms in multinational cohorts and across various clinical environments.

AI technologies, such as machine learning algorithms, image recognition systems, and natural language processing tools, offer the ability to analyze large datasets from various sources (*e.g*., imaging, genomics, and patient records) [54, 78-80] and generate insights that would be difficult or time-consuming for humans to identify. The increase in AI clinical trials for CNS diseases since 2017 is notable, with the number of trials rising to nine per year by 2023. The inclusion of AI in the management of CNS diseases, such as stroke, SCI, and TBI, reflects the increasing recognition of AI’s potential to address the complexities of neurological conditions. However, 51 clinical trials were included in this study; only 11 reported outcome data [66, 71, 73, 81-88]. Given the lack of methodological and clinical homogeneity across these studies, a quantitative meta-analysis was not feasible. Therefore, a qualitative synthesis was performed to summarize the available evidence. Overall, the findings suggest potential benefits of AI applications in CNS disorders. Future studies should adopt more standardized designs to generate homogeneous evidence and provide comprehensive descriptions of the AI workflow, including algorithm selection, input variables, model training and validation, and clearly reported performance metrics.

Another key finding in this study was the underrepresentation of PROMs in AI clinical trials. PROMs provide crucial information on how patients perceive their symptoms, quality of life, and overall health status, offering insights that clinical measures alone cannot capture [67, 89-91]. Despite the increasing emphasis on PROMs in healthcare research, the analysis revealed that only about one-fourth of the included trials incorporated such measures. One potential reason may be the prioritization of objective clinical endpoints (*e.g*., imaging and biomarkers) over subjective patient experiences, particularly in early-stage AI studies where model performance is the main focus [83, 86-88]. Additionally, the integration of PROMs into AI systems poses methodological challenges, such as standardizing unstructured textual data and accounting for inter-individual variability. In some cases, limited clinician awareness of the value of PROMs or insufficient infrastructure for data collection may also hinder their use [92]. Addressing these issues will require increased awareness, standardized PROMs integration frameworks, and greater emphasis on patient-centered outcomes in future AI research.

Notably, among the 51 trials included in this study, only 13 were rated as high quality based on the risk of bias assessment, highlighting a general lack of methodological rigor and transparency in this field. Furthermore, the lack of phase information in most clinical trials suggests that many AI-based interventions are still in early stages of development and remain far from achieving the level of evidence required for phase IV studies. In addition to these limitations, several practical barriers may hinder the implementation of AI in CNS clinical trials. One major concern is data privacy and security, especially given the sensitive nature of neurological patient data, such as brain imaging, genetic profiles, and behavioral records [93, 94]. Another critical concern is clinician acceptance. Many healthcare professionals express skepticism toward AI-driven decision-making, citing concerns about model interpretability, ambiguous legal liability, and potential disruption of established clinical workflows [95]. Overcoming these barriers will be essential for the successful translation of AI innovations from research settings to routine clinical practice in CNS disease management.

This study provides a comprehensive overview of the current status and trends in AI applications for CNS injury-related clinical trials, highlighting both technological advancements and existing limitations. By identifying the dominance of early-phase studies and the insufficient incorporation of patient-centered tools like PROMs, this work emphasizes the need to align AI development with real-world clinical and patient-reported outcomes [96-114]. These insights may guide researchers and clinicians in designing future studies that not only optimize algorithmic performance but also focus on the holistic improvement of patient well-being, thereby fostering more responsible and impactful AI integration into healthcare.

## CONCLUSION

In conclusion, AI clinical trials in CNS injuries are rapidly increasing, with notable progress in conditions, such as stroke, SCI, and TBI. While AI shows great promise in enhancing treatment, diagnosis, and patient care, the limited use of PROMs suggests a gap in capturing patient-centered outcomes. To advance the field, future work should move beyond technological validation toward clinical integration, personalization, and long-term efficacy. This includes incorporating PROMs systematically to evaluate how AI interventions affect symptoms, function, and quality of life, as well as exploring multi-source data fusion, such as neuroimaging and sensor-based monitoring, to improve decision-making across the continuum of care. Furthermore, a comprehensive topic analysis of AI-related CNS trials could identify key research themes, target populations, and methodological trends, guiding the development of standardized frameworks for evaluating AI interventions across all stages of CNS injuries, from acute management to long-term rehabilitation. By bridging these gaps and embracing a broader, forward-looking research agenda, AI clinical trials in CNS injuries can evolve from early experimentation to impactful real-world applications, ultimately benefiting both patients and the healthcare system.

## Figures and Tables

**Table 1:** Characteristics of clinical trials on AI in CNS diseases.

**Records**	**Number of ** **Clinical Trials**	**Country**	**CNS Diseases**	**Study ** **Design**	**Age of Participants (Years)**	**Sample Sizes**	**Purpose**	**AI ** **Approach/** **Algorithm**	**Control Group/** **Reference Group**	**Performance Metrics**	**Patient-reported Outcomes**
1	NCT05838456	USA	Ischemic Stroke	Single-center trial	> 18	443	Assess the effect of incorporating Viz. AI software for endovascular stroke therapy screening	AI-based automated detection	NA	NA	NA
2	NCT05903898	Norway	Ischemic Stroke	Non-randomized, parallel study	> 18	1000	Evaluate whether the AI support tool (StrokeSens, Circle NVI) can accelerate decision-making and improve detection rates in patients with acute ischemic stroke due to intracranial large vessel occlusion (LVO) or medium vessel occlusion	AI software StrokeSens (Circle NVI)	NA	NA	NA
3	NCT06817564	China	Traumatic Brain Injury	Single-center parallel RCT	> 18	125	Evaluate the feasibility, acceptability, and preliminary effectiveness of the AI-driven Personalized Exercise Feedback Program (AI-PEF) in improving outcomes for patients with traumatic brain injury.	AI-PEF	NA	NA	Revised Sport Motivation Scale-II (SMS-II), Rivermead Post-Concussion Symptoms Questionnaire (RPQ), Beck Depression Inventory-II (BDI-II), Pittsburgh Sleep Quality Index (PSQI), Godin-Shephard Leisure Time Physical Activity Questionnaire (GLTPA), World Health Organization Quality of Life Scale-brief (WHOQOL-BREF), and Mobile App Usability Questionnaire (MAUQ)
4	NCT06645405	China	Ischemic Stroke	Stepped-wedge, cluster RCT	18-95	174	Develop an AI algorithm to automate the reconstruction of computed tomography (CT) angiography	AI algorithm	NA	NA	NA
5	NCT05437237	Netherlands	Ischemic Stroke	Single-center trial	> 18	1192	Develop novel AI-based algorithms with optimal diagnostic accuracy for the identification of LVO stroke in patients with a suspected acute ischemic stroke	AI-STROKE algorithm	NA	NA	NA
6	NCT06453733	UK	Traumatic Brain Injury	Single-center trial	16-99	54	Advance the Crainio device, designed for non-invasive intracranial pressure (ICP) monitoring	Crainio machine learning algorithm	NA	NA	NA
7	NCT01608438	USA	Spinal Cord Injury	Non-randomized, parallel study	18-65	157	Develop personalized interfaces based on wearable sensors that enable paralyzed persons to control computers, wheelchairs, and other assistive devices	Machine learning algorithm	NA	NA	NA
8	NCT04524624	China	Ischemic Stroke	Single-center, parallel RCT	18-90	21689	Evaluate the effectiveness of an AI-based clinical decision support system for stroke management in patients with acute ischemic stroke within 7 days of symptom onset	AI and clinical decision support system	NA	NA	NA
9	NCT04447599	France	Ischemic Stroke	Single-center trial	55-80	850	Evaluate a correlation between abnormalities of conjunctival vessels and the staging of small cerebral disease	Deep learning	NA	NA	NA
10	NCT04302259	USA	Spinal Cord Injury	Single-center trial	18-65	3	Develop an intelligent spine interface (ISI) that interprets and transfers neural information from spinal cord injury	Artificial neural network-based computer interpreter	NA	NA	NA
11	NCT05745051	Israel	Ischemic Stroke	Single-center trial	> 18	100	Evaluate the safety and effectiveness of the CVA-FLOW software device for acute ischemic stroke	CVA-FLOW, a digital health AI-based Telestroke system	NA	NA	NA
12	NCT05874596	China	Ischemic Stroke	Randomized, parallel study	> 18	1221	Evaluate the improvement of patients with acute ischemic stroke undergoing endovascular treatment based on an AI-aided clinical feedback system	AI-aided clinical feedback system	NA	NA	NA
13	NCT05466864	China	Ischemic Stroke	Single-center trial	18-80	120	Screening obstructive sleep apnea in hospitalized patients admitted for acute ischemic stroke	A medical-grade wearable with a neural network algorithm	NA	NA	NA
14	NCT06828679	China	Ischemic Stroke	Randomized, parallel study	65-80	400	Build personalized rehabilitation pathways for maximizing motor functional recovery	AI-powered prediction system	Group receiving typical post-stroke care	NA	NA
15	NCT05959746	France	Ischemic Stroke	Single-center trial	> 18	300	Develop a tool for optimized self- and hetero-diagnosis of stroke	AI-STROKE application	NA	NA	NA
16	NCT05670873	China	Ischemic Stroke	Randomized, parallel study	18-80	50	Evaluate the potential of transcranial magnetic stimulation in patients with acute severe ischemic stroke and disorder of consciousness	Hybrid neural network	NA	NA	NA
17	NCT06183970	Russia	Ischemic or hemorrhagic stroke	Randomized, parallel study	18-80	90	Assess the recovery of motor functions in patients with cerebral stroke	AI-driven video analysis and machine learning	NA	NA	NA
18	NCT06334796	Spain	Multiple diseases, including stroke	Single-center trial	> 18	10	Evaluate the performance of an AI-powered virtual assistant for emergency neurological triage.	AI-powered virtual assistant	NA	NA	Satisfaction scale
19	NCT03865979	USA	Intracerebral Hemorrhage	Non-randomized, multi-center trial	> 18	10	Evaluate the performance of the Viz RECRUIT software in detecting patients with intracerebral hemorrhage.	Viz RECRUIT software	NA	NA	No
20	NCT04457908	China	Silent Cerebrovascular Disease	Multi-center, randomized, double-blind, parallel-controlled, prospective study	60-85	1000	Evaluate the effectiveness and cost-effectiveness of intelligent and doctor groups for gait disorder screening	AI system	Group receiving the neurological function assessment from the doctor.	NA	No
21	NCT05214521	Russia	Ischemic Stroke	Single-center trial	18-70	30	Evaluate the effectiveness and safety of rehabilitation of patients with impaired fine motor function of the hand	Glove simulator “SensoRehab” based on digital interactive technologies with AI	NA	NA	No
22	NCT04058379	France	Traumatic Brain Injury	Single-center trial	> 18	30	AI analysis of the initial scan evolution of traumatic brain injury patients to predict neurological outcome	NA	NA	NA	No
23	NCT02599259	USA	Ischemic Stroke	Randomized, parallel study	> 18	28	Measure and optimize adherence in patients on anticoagulation therapy using AI on mobile devices	AiCure Platform	Group receiving treatment as usual	NA	No
24	NCT06465719	China	Intracerebral Hemorrhage	Randomized, parallel study	> 18	312	Evaluate the therapeutic effects of the fiber tract-based AI Robot Guiding System on the perioperative and long-term recovery of patients with small-volume basal ganglion hemorrhage	Tract-based AI Robot Guiding System	NA	NA	No
25	NCT06783049	China	Ischemic Stroke	Randomized, parallel study	> 18	4490	Evaluate the effectiveness of the secondary prevention of ischemic stroke	A digital health-based intelligent management system	The control group received no additional intervention for secondary prevention	NA	Fatigue severity scale
26	NCT05391919	Russia	Ischemic Stroke	Single-center trial	45-75	90	Evaluate the effectiveness and safety of multimodal rehabilitation technology.	Digital technologies, biofeedback, virtual reality, and neurointerface technology	Group without the intervention of multimodal technology	NA	Hospital anxiety and depression scale
27	NCT02833428	France	Spinal Cord Injury	Single-center trial	> 18	1	Analyze the influence of the physiopathological environment of the traumatized spinal cord on spinal pain using an AI engine	NA	NA	NA	No
28	NCT04039178	Israel	Ischemic Stroke	Prospective, randomized, double-blinded, sham device-controlled study	18-80	28	Evaluate the efficacy of the Electromagnetic field-based brain-computer interface device in the management of acute stroke patients.	BrainQ stimulation device based on machine learning	Sham BrainQ treatment	NA	PROMIS global health short form
29	NCT03452059	China	Spinal Cord Injury	Prospective, multi-center trial	18-60	40	Evaluate the effectiveness and safety of AI-robotic-assisted lower extremity exoskeleton rehabilitation training in patients with spinal cord injury	AiLegs/ AiWalker	NA	NA	No
30	NCT06538506	Canada	Ischemic or hemorrhagic stroke	Single-center trial	> 18	60	Evaluate the effect of RehUp brain computer interface (BCI) therapy in the chronic phase of stroke with moderate to severe upper extremity impairment.	VIBRAINT BCI robot	Control therapy without BCI intervention	NA	Patient health questionnaire and euroQuol measure of health status (Version 5D)
31	NCT06534164	UK	Multiple diseases, including stroke	Randomized, parallel study	40-80	460	Assess the effectiveness of the telerehabilitation of the balance clinical and economic decision support system	TeleRehab DSS system	Control group receiving standard balance rehabilitation	NA	Visual analogue scale, activities-specific balance confidence scale, hospital anxiety and depression scale, fatigue severity scale, Warwick-Edinburgh mental Wellbeing scale, situational vertigo questionnaire, and eHealth literacy assessment
32	NCT05944666	Russia	Ischemic Stroke	Randomized, parallel study	45-75	405	Develop a standardized multimodal cognitive-motor rehabilitation system for post-stroke patients	Multimodal technology	NA	NA	The 36-item short form health survey
33	NCT05238389	Italy	Ischemic or hemorrhagic stroke	Single-center trial	18-85	44	Develop an AI-based upper limb robotic therapy for stroke patients	Decision support system	Control group without intervention	NA	Numeric rating scale (NRS) for pain and 36-item short form health survey
34	NCT06452888	China	Ischemic Stroke	Multi-center trial	18-80	3540	Improve medical quality and outcomes through an intelligent management and decision system for cerebrovascular diseases	Clinical decision support system	NA	NA	No
35	NCT06242938	China	Intracerebral Hemorrhage	Randomized, parallel study	> 18	680	Evaluate the efficacy, safety, and feasibility of early intensive antihypertensive treatment in intracerebral hemorrhage (ICH) patients at high risk of hematoma expansion based on AI prediction.	NA	Control group with standard antihypertensive treatment	NA	EuroQol-5 dimensions (EQ-5D) score
36	NCT04957862	China	Intracerebral Hemorrhage	Randomized, parallel study	18-75	428	Accelerate functional rehabilitation using robot-assisted evacuation of subacute and chronic supratentorial deep hypertensive intracerebral hemorrhage.	NA	Group receiving standard medical treatment	NA	No
37	ChiCTR2200060214	China	Ischemic or hemorrhagic stroke	Single-center, single-blind RCT	20-70	50	Evaluate the upper limb motor function for poststroke patients	Virtual reality (VR) rehabilitation training system	Control group without VR training	NA	Participant-reported questionnaire on usability and satisfaction
38	NCT05978700	China	Ischemic or hemorrhagic stroke	Prospective, single-blinded RCT	> 18	84	Evaluate the post-stroke dysphagia	AI-based video-game (VG) system including three games: “Collecting Carrots”, “Out of the Maze”, and “Leaping Barriers”	Usual care group without an AI-based VG system	The AI-VG group significantly improves swallowing function post-stroke	Participant-reported questionnaire on usability and satisfaction
39	CRIS (KCT0009013)	Korea	Ischemic or hemorrhagic stroke	Double-blinded, parallel-group RCT	> 19	25	Evaluate the efficacy of brain-computer interface (BCI) training with motor imagery-contingent feedback on upper limb function and neuroplasticity in individuals with chronic stroke.	The recovery X-PRO comprises an electroencephalogram, functional electrical stimulation, and a computer screen projecting virtual hands	Motor imagery (MI) -independent feedback BCI group	MI-contingent BCI training enhances motor function and neuroplasticity in chronic stroke	Self-reported questionnaire for persons with stroke
40	NCT06045455	Czechia	Ischemic or hemorrhagic stroke	Prospective observational multicentre study	> 18	Expected enrollment1: 3,000 participants	Evaluate the specificity and sensitivity of multimodal CT in the diagnosis of stroke mimics versus stroke	Multimodal brain CT including non-contrast head CT, CT perfusion, and CT angiography	Patients with stroke mimics	NA	NA
41	NCT06027411	UK	Multiple diseases, including stroke	Multicentre stepped-wedge cluster-randomised study	> 18	Expected total number of scans: 16,800	Evaluate Qure.ai’s qER software in identifying and prioritising patients with critical findings from AI analysis of non-contrast head CT scans	qER EU 2.0 software developed using deep-learning algorithm	Patients without qER processed scans	NA	NA
42	CHiCTR1800017568	China	Ischemic or hemorrhagic stroke	Within-subject comparative study	63.8 ± 14.8	20	Classify post-stroke hand gesture	One-shot transfer learning using prototypical networks and neural networks	Conventional transfer learning, neural networks, and traditional machine learning approaches	Accuracy: 82.2%	NA
43	jRCTs032200045	Japan	Ischemic or hemorrhagic stroke	Single-blinded RCT	20-80	20	Assess the effect of an AI-integrated electromyography (EMG) –driven robot hand in patients with chronic stroke	AI-integrated EMG-driven robot	The control group performing passive finger exercises provided by an AI-integrated EMG-driven robot	The AI-integrated EMG-driven robot produced lasting improvements in upper extremity motor function and spasticity for up to 4 weeks	NA
44	NCT03241732	USA	Ischemic or hemorrhagic stroke	Single-center clinical trial	> 18	100	Differentiate patients with chronic mild traumatic brain injury (mTBI) from healthy controls.	The support vector machine (SVM) models were developed based on resting-state functional magnetic resonance imaging (rs-fMRI) metrics, such as fractional amplitude of low-frequency fluctuation, regional homogeneity, degree centrality, voxel-mirrored homotopic connectivity, functional connectivity strength, and seed-based functional connectivity.	Healthy controls	Operating characteristic curve (AUC) among local measures: 86.67-100%; AUC among network measures: 80.42-93.33%	NA
45	NCT00912041	USA	Multiple diseases, including stroke	Prospective, open-label, nonrandomized clinical trial	18-75	14	Evaluate the interim safety of the BrainGate neural interface system implanted in patients with paralysis.	BrainGate neural interface system	NA	Sixty-eight device-related adverse events during implantation, with no safety events requiring explantation	NA
46	RBR-2pb8gq	Brazil	Spinal cord injury	Parallel two-arm randomized pilot study	NA	8	Investigate the effect of assisted locomotion training combined with noninvasive brain machine interfaces (BMI) and tactile feedback on promoting partial neurological recovery in individuals with the most severe sensory and motor deficits following spinal cord injury	Noninvasive brain-machine interface, virtual reality, and tactile feedback	Patients with intensive assisted locomotion training	Locomotion training combined with BMI enhanced both the recovery rate and the BMI classifier accuracy	NA
47	NCT04620707	Spain	Hemiparesis post-stroke	Randomized longitudinal clinical trial	20-85	90	Compare the effects of the intervention using the Rehabilitation Gaming System (RGS) platform combined with standard care versus standard care alone.	A cloud-based platform for AI-enhanced rehabilitation, integrating virtual reality, motion capture, and wearable technologies.	The control group receives standard care without RGS intervention	NA	NA
48	NA	China	Intracerebral Hemorrhage	Non-controlled retrospective pilot clinical trial	NA	2999	Validate the diagnostic efficacy of the DeepCT system in patients with intracerebral hemorrhage	DeepCT: a deep convolutional neural network-based framework	NA	Sensitivity of 96.453%, specificity of 99.52%, and accuracy of 99.16%	NA
49	NCT01274117	China	Ischemic Stroke	Parallel RCT	> 18	21897	Identify the cause of ischemic stroke by machine learning and evaluate its accuracy	Six machine learning models: Random Forests (RF), Logistic Regression (LR), Extreme Gradient Boosting (XGBoost), K-Nearest Neighbors (KNN), AdaBoost, and Gradient Boosting Machine (GBM)	NA	RF is the best model, achieving AUC values of 0.981, 0.919, and 0.918 for predicting cardioembolism, large-artery atherosclerosis, and small-artery occlusion, respectively	NA
50	NA	USA	Intracerebral Hemorrhage	Multicenter RCT	18-80	500	Evaluate the performance and clinical applicability of deep learning-based intracerebral hemorrhage segmentation tools	DeepBleed: 3-dimensional deep neural network	NA	DeepBleed achieves accurate segmentation of intracerebral hemorrhage, with a best mean and median Dice coefficient of 0.914 and 0.919, and a volume correlation of 0.979	NA
51	NA	Netherlands	Ischemic Stroke	Multicenter RCT	> 18	1383	Evaluate the predicting outcome of endovascular treatment for acute ischemic stroke	RF, SVM, Neural Network, and Super Learner	NA	No superior performance of machine learning algorithms over logistic regression models in predicting reperfusion and 3-month functional independence after endovascular treatment	NA

## Data Availability

All data generated or analyzed during this study are included in this published article.

## LIST OF ABBREVIATIONS

AI: Artificial Intelligence
CNS: Central Nervous System
CSV: Comma-separated Values
DL: Deep Learning
HIE: Hypoxic-ischemic Encephalopathy
LLM: Large Language Model
ML: Machine Learning
NCT: National Clinical Trial
NLP: Natural Language Processing
PROMs: Patient-reported Outcome Measures
SCI: Spinal Cord Injury
TBI: Traumatic Brain Injury

## STANDARDS OF REPORTING

PRISMA guidelines and methodology were followed.

## SUPPLEMENTARY MATERIAL

PRISMA checklist is available as supplementary material on the publisher’s website along with the published article.
